# *In vitro* modeling to determine mutation specificity of EGFR tyrosine kinase inhibitors against clinically relevant *EGFR* mutants in non-small-cell lung cancer

**DOI:** 10.18632/oncotarget.5887

**Published:** 2015-10-15

**Authors:** Toshiyuki Hirano, Hiroyuki Yasuda, Tetsuo Tani, Junko Hamamoto, Ayano Oashi, Kota Ishioka, Daisuke Arai, Shigenari Nukaga, Masayoshi Miyawaki, Ichiro Kawada, Katsuhiko Naoki, Daniel B. Costa, Susumu S. Kobayashi, Tomoko Betsuyaku, Kenzo Soejima

**Affiliations:** ^1^ Division of Pulmonary Medicine, Department of Medicine, Keio University, School of Medicine 35 Shinanomachi, Shinjuku-ku, Tokyo, Japan; ^2^ Keio Cancer Center, Keio University, School of Medicine 35 Shinanomachi, Shinjuku-ku, Tokyo, Japan; ^3^ Division of Hematology/Oncology, Beth Israel Deaconess Medical Center, Harvard Medical School, Boston, MA, USA

**Keywords:** EGFR mutation, EGFR tyrosine kinase inhibitors, EGFR exon 20 insertion mutations, in vitro modeling, lung cancer

## Abstract

*EGFR* mutated lung cancer accounts for a significant subgroup of non-small-cell lung cancer (NSCLC). Over the last decade, multiple EGFR tyrosine kinase inhibitors (EGFR-TKIs) have been developed to target mutated EGFR. However, there is little information regarding mutation specific potency of EGFR-TKIs against various types of *EGFR* mutations. The purpose of this study is to establish an *in vitro* model to determine the “therapeutic window” of EGFR-TKIs against various types of *EGFR* mutations, including EGFR exon 20 insertion mutations. The potency of 1^st^ (erlotinib), 2^nd^ (afatinib) and 3^rd^ (osimertinib and rociletinib) generation EGFR-TKIs was compared *in vitro* for human lung cancer cell lines and Ba/F3 cells, which exogenously express mutated or wild type *EGFR*. An *in vitro* model of mutation specificity was created by calculating the ratio of IC50 values between mutated and wild type EGFR. The *in vitro* model identified a wide therapeutic window of afatinib for exon 19 deletions and L858R and of osimertinib and rociletinib for T790M positive mutations. The results obtained with our models matched well with previously reported preclinical and clinical data. Interestingly, for *EGFR* exon 20 insertion mutations, most of which are known to be resistant to 1^st^ and 2^nd^ generation EGFR-TKIS, osimertinib was potent and presented a wide therapeutic window. To our knowledge, this is the first report that has identified the therapeutic window of osimertinib for *EGFR* exon 20 insertion mutations. In conclusion, this model will provide a preclinical rationale for proper selection of EGFR-TKIs against clinically-relevant *EGFR* mutations.

## INTRODUCTION

Lung cancer is the leading cause of cancer related death worldwide [1]. The identification of somatic mutations within the epidermal growth factor receptor (*EGFR*) kinase domain helped our understanding of the biology of lung cancer harboring *EGFR* mutations [2–6]. *EGFR* mutations are expected to activate the EGFR by destabilizing the inactive form of EGFR without ligand stimulation [7–9]. Activated EGFR induces EGFR-mediated pro-survival and anti-apoptotic signals through downstream targets such as extracellular-signal-regulated kinase (ERK)/mitogen-activated protein kinase (MAPK) and phosphatidylinositol-3-kinases (PI3K)/protein kinase B (AKT) [10, 11]. Inhibition of the EGFR pathway leads to the down-regulation of pro-survival signals and up-regulation of pro-apoptotic molecules [12], by which EGFR tyrosine kinase inhibitors (EGFR-TKIs) exert their dramatic effects in patients with *EGFR* mutated lung cancer.

*EGFR* mutations have been identified in approximately 10–30% of non-small-cell lung cancer (NSCLC) [13, 14]. The most common, “classic” mutations are in-frame deletions around the LREA motif of exon 19 (approximately 45% of *EGFR* mutations) and the exon 21 L858R point mutation (approximately 40% of *EGFR* mutations). Other relatively rare *EGFR* mutations include, G719X (3% of *EGFR* mutations) and L861Q (2% of *EGFR* mutations) [10]. Another main group of *EGFR* mutations include exon 20 insertion mutations (4–10% of *EGFR* mutations) [15, 16].

EGFR tyrosine kinase inhibitors (EGFR-TKIs) have been developed to target mutated EGFR. EGFR-TKIs reversibly or irreversibly bind to the ATP binding pocket of EGFR and inhibit the phosphorylation of EGFR, thereby inhibiting the activation of the EGFR signaling pathway. The exon 19 deletions, L858R, G719X, and L861Q mutations are 1^st^ generation EGFR-TKIs, gefitinib and erlotinib, sensitizing mutations. The response rates to gefitinib or erlotinib are around 60–80% [14, 17].

Most *EGFR* exon 20 insertion mutations are 1^st^ generation EGFR-TKIs resistant mutations [15, 18, 19]. One exception is *EGFR* A763_Y764insFQEA, which we previously reported as another 1^st^ generation EGFR-TKIs sensitizing mutation [20]. For these 1^st^ generation EGFR-TKIs resistant exon 20 insertion mutations, no potent inhibitor has been reported. Therefore, patients with NSCLC harboring *EGFR* exon 20 insertion mutations present a shorter survival time compared to patients with classic *EGFR* mutations [21]. The development of EGFR-TKIs, which effectively inhibit EGFR with exon 20 insertions, but not the wild type EGFR, has been anticipated.

The 1^st^ generation reversible EGFR-TKIs, gefitinib and erlotinib, dramatically changed the treatment strategy for patients harboring *EGFR* mutated lung cancer. The significant benefit of gefitinib or erlotinib for patients with NSCLC harboring EGFR-TKIs sensitizing mutations was repeatedly demonstrated in multiple clinical trials [22, 23]. However, despite the initial favorable response, lung cancer cells eventually acquire resistance to gefitinib or erlotinib. *EGFR* T790M mutations account for about 50% of acquired resistance to gefitinib or erlotinib [24, 25]. To target *EGFR* mutations, including *EGFR* T790M mutation, multiple EGFR-TKIs have been developed. These include 2^nd^ generation EGFR-TKIs, afatinib [26] and dacomitinib [27, 28], as well as 3^rd^ generation EGFR-TKIs, WZ4002 [29], osimertinib (formerly AZD9291) [30, 31] and rociletinib [32, 33].

Afatinib, a clinically available 2^nd^ generation EGFR-TKI, is potent against *EGFR* mutated lung cancer cells *in vitro* [26] and *in vivo* [34, 35]. However, for *EGFR* T790M mutated lung cancer, it failed to overcome EGFR T790M-mediated resistance in patients [36, 37]. Osimertinib and rociletinib are 3^rd^ generation EGFR-TKIs, both of which are reported to be effective in lung cancer cells harboring *EGFR* T790M in preclinical models [30, 32]. Promising results of phase I/II study of osimertinib and rociletinib have recently been published. Osimertinib showed a promising safety and efficacy, the response rate and progression free survival for *EGFR* T790M positive patients was 61% and 9.6 months, respectively [38]. Similarly, the response rate of rociletinib for *EGFR* T790M positive patients was 59% [39].

Today, we have multiple EGFR-TKI options to treat patients with lung cancer harboring *EGFR* mutations. However, there is no clear guideline regarding which EGFR-TKIs should be used for which mutation. To solve this problem and provide a model, which clinicians or physician scientists could refer to, we examined and compared the potency of EGFR-TKIs against lung cancer cell lines harboring various types of *EGFR* mutations. In addition, we created an *in vitro* model, which allows us to determine the therapeutic window of EGFR-TKIs. This model will provide a preclinical rationale for proper selection of EGFR-TKIs against clinically-relevant *EGFR* mutations.

## RESULTS

### Comparison of the sensitivity/resistance profile of Ba/F3 stable cell lines

In order to directly compare the sensitivity of multiple *EGFR* mutations to EGFR-TKIs, we generated *EGFR* transduced Ba/F3 stable cell lines. Wild type and mutated *EGFR* were exogenously transduced into Ba/F3 cells. The sensitivity to EGFR-TKIs was evaluated by MTS assay with or without EGFR-TKIs.

**Figure 1:**
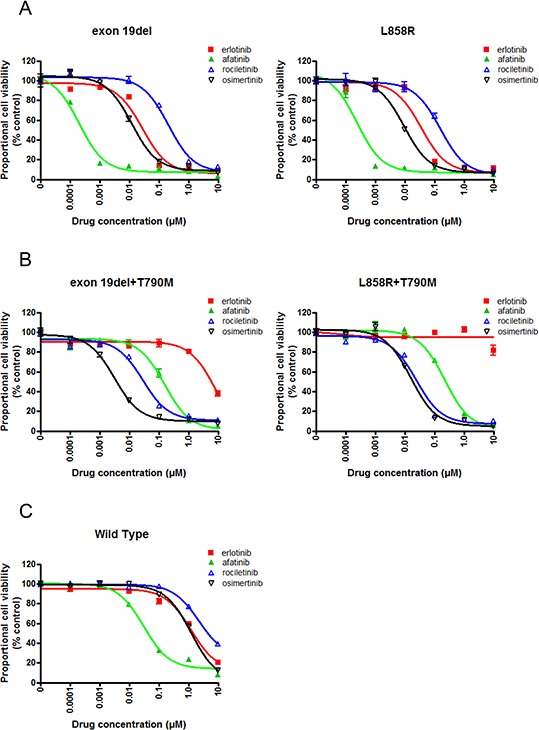
Sensitivity of Ba/F3 cells harboring *EGFR* mutations to EGFR-TKIs **A.** MTS assay for Ba/F3 cells harboring *EGFR* exon 19 deletion and L858R. The proportional cell viability is shown. **B.** MTS assay for Ba/F3 cells harboring *EGFR* exon 19 deletion+T790M and L858R+T790M. The proportional cell viability is shown. **C.** MTS assay for Ba/F3 cells harboring wild type *EGFR*. The proportional cell viability is shown. Erlotinib, afatinib, osimertinib, and rociletinib were used as EGFR-TKIs. Error bars indicate standard deviation.

Cell growth of Ba/F3 cells harboring classic *EGFR* mutations, exon 19 deletion (exon 19del) and L858R, was dramatically inhibited by afatinib (Figure 1A). Although less potent than afatinib, erlotinib effectively inhibited the proliferation of Ba/F3 cells. The potency of osimertinib was comparable to that of erlotinib, while rociletinib was less potent than erlotinib and osimertinib.

Erlotinib did not inhibit the proliferation of Ba/F3 cells harboring *EGFR* T790M mutations, EGFR exon 19del+T790M, and L858R+T790M, at low concentrations. However, afatinib and 3^rd^ generation EGFR-TKIs, osimertinib and rociletinib, effectively inhibited the proliferation of Ba/F3 cells (Figure 1B).

In the clinic, skin rash, interstitial pneumonia, or diarrhea are common or life threatening side effect of EGFR-TKIs. These side effects most likely due to the inhibition of the wild type EGFR expressed in the epithelial cells present in the skin, lungs, and gastrointestinal tract by EGFR-TKIs. Hence, EGFR-TKIs highly selective to mutated EGFR, but not to wild-type EGFR, can efficiently inhibit mutated EGFR in lung cancer cells without affecting the wild type EGFR expressed in epithelial cells. To evaluate the effect of EGFR-TKIs on the wild type EGFR, we performed an MTS assay in the presence of EGF and with or without EGFR-TKIs (Figure 1C). Ba/F3 cells were efficiently inhibited by afatinib. The IC50 value of afatinib was 31 nM, whereas those of the other three EGFR-TKIs were comparable, indicating that afatinib was the most potent EGFR-TKI against the wild type EGFR.

**Figure 2:**
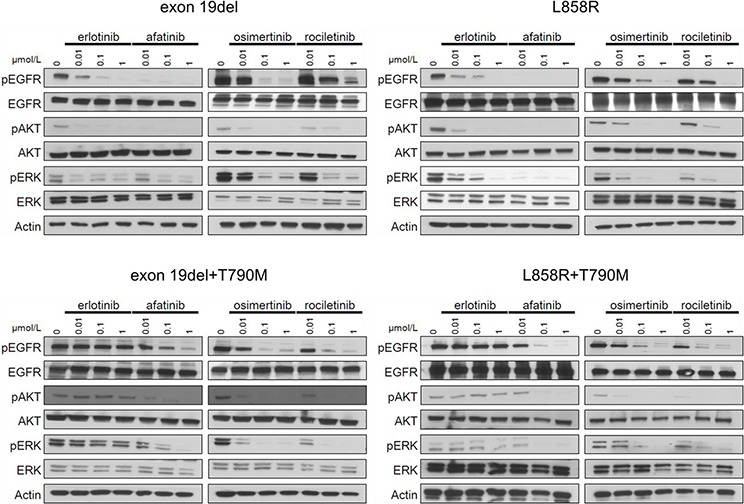
Inhibition of the phosphorylation of EGFR and downstream proteins by EGFR-TKIs in BaF3 cells harboring *EGFR* mutations The results of immunoblotting for Ba/F3 cells with *EGFR* exon 19 deletion, L858R, exon 19 deletion+T790M, and L858R+T790M are shown. The cells were treated with the indicated concentrations of EGFR-TKIs for 4 h. Erlotinib, afatinib, osimertinib, and rociletinib were used as EGFR-TKIs. pEGFR, pAKT, and pERK indicate the phosphorylated form of EGFR, AKT, and ERK, respectively. Actin was used as a loading control.

Immunoblotting was then performed to examine whether the aforementioned effect of EGFR-TKIs on Ba/F3 cells was exerted through inhibition of the EGFR signaling pathway (Figure 2).

In Ba/F3 cells harboring classic *EGFR* mutations, exon 19 deletions and L858R, afatinib dramatically inhibited the phosphorylation of EGFR, AKT, and ERK. Erlotinib and osimertinib comparably inhibited the phosphorylation of EGFR, AKT, and ERK. Erlotinib did not inhibit the phosphorylation of EGFR, AKT, and ERK in Ba/F3 cells harboring *EGFR* T790M mutations, exon 19del+T790M, and L858R+T790M. However, afatinib and 3^rd^ generation EGFR-TKIs, osimertinib and rociletinib, effectively inhibited the phosphorylation of EGFR, AKT, and ERK. These data indicate that the sensitivity of Ba/F3 cells to EGFR-TKIs was mediated through inhibition of the EGFR and downstream signals.

### Establishment of an *in vitro* model to determine the therapeutic window of EGFR-TKIs

**Figure 3:**
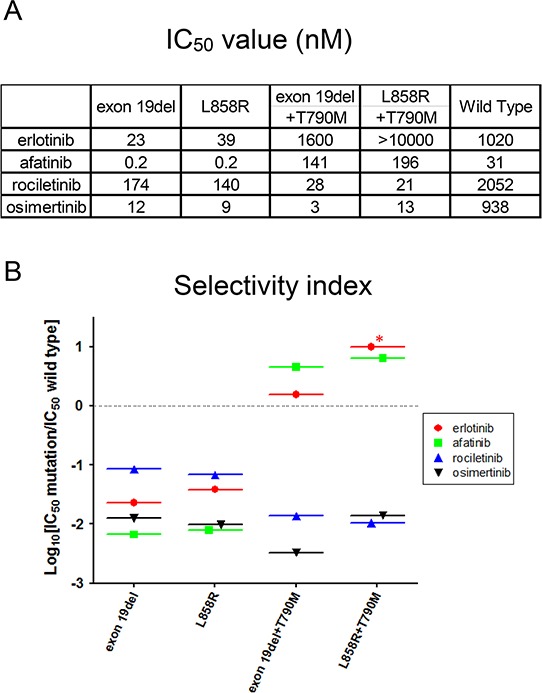
IC50 values and *in vitro* modeling **A.** IC50 values (nM) of EGFR-TKIs for wild type and mutated *EGFR* are shown. Erlotinib, afatinib, osimertinib, and rociletinib were used as EGFR-TKIs. **B.** Calculated values of the selectivity index (SI) for *EGFR* mutations, exon 19 deletion, L858R, exon 19 deletion+T790M, and L858R+T790M. *; SI index >1.

The IC50 values of all EGFR-TKIs examined in this study are summarized in Figure 3A. Afatinib is reported to be ineffective against lung cancer harboring EGFR T790M positive mutations, because concentrations at which afatinib inhibits lung cancer cells harboring EGFR T790M are not achievable in humans due to dose limiting toxicities such as skin rash and diarrhea, indicating a narrow therapeutic window of afatinib for *EGFR* T790M [40].

To provide an estimation of the therapeutic window of EGFR-TKIs, we created an *in vitro* model by calculating the ratio of IC50 values of the wild type *EGFR* and mutated *EGFR*, referred to as the selectivity index (SI) (Figure 3B). In this model, the selectivity of each EGFR-TKI is high if the SI value is low. For Ba/F3 cells harboring classic *EGFR* mutations, exon 19 deletions and L858R, afatinib showed the lowest SI values among EGFR-TKIs studied in this study, indicating a wide therapeutic window of afatinib for these mutations. Interestingly, for Ba/F3 cells harboring classic *EGFR* mutations, osimertinib showed mutation specificity similar to that of afatinib, and the SI values were around −2, indicating IC50 values for these classic mutations were about 100-fold lower than those for the wild-type *EGFR*.

For Ba/F3 cells harboring *EGFR* T790M, 3^rd^ generation EGFR-TKIs, osimertinib and rociletinib, showed striking mutation specificity. The SI values were around −2 or lower, indicating IC50 values for T790M mutation more than 100-fold lower than those for the wild-type *EGFR*. Unfortunately, erlotinib and afatinib did not demonstrate mutation specificity for *EGFR* T790M mutations. The SI values were more than zero, indicating that the IC50 values of erlotinib and afatinib for *EGFR* T790M mutations were higher than those for the wild-type *EGFR*.

To determine and to confirm the clinical relevance of this *in vitro* model, we reviewed the data of human clinical trials. The efficacy of erlotinib for classic *EGFR* mutations [41, 42] and inefficacy of erlotinib for T790M mutations are reported [24] and well accepted. Our *in vitro* model clearly indicated the difference of erlotinib efficacy for classic *EGFR* mutations and its inefficacy for T790M mutations. Again, the efficacy of afatinib for classic *EGFR* mutations [43] and its inefficacy for T790M mutations are reported [36]. Data from our *in vitro* model were in agreement with these results. The efficacy of osimertinib and rociletinib for patients with NSCLC harboring EGFR T790M mutation and little dose limiting toxicities have recently been described. The striking mutation specificity of osimertinib and rociletinib shown in the model matches well with the results of the reported trials. In summary, the results of our *in vitro* model match well with the reported clinical trials.

### Confirmation of the sensitivity/resistance profile of EGFR-TKIs in lung cancer cell lines

**Figure 4:**
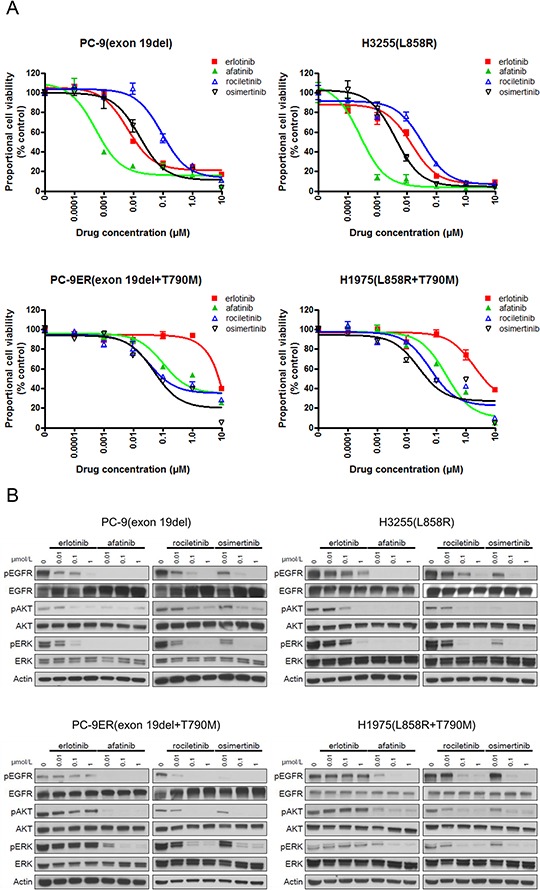
Sensitivity of lung cancer cell lines to EGFR-TKIs **A.** MTS assay for PC-9, H3255, PC9-ER, and H1975 cells. The IC50 values (nM) for EGFR-TKIs are shown. Error bars indicate standard deviation. **B.** The results of immunoblotting for PC-9, H3255, PC9-ER, and H1975 cells are shown. The cells were treated with the indicated concentrations of EGFR-TKIs for 4 h. Erlotinib, afatinib, osimertinib, and rociletinib were used as EGFR-TKIs. pEGFR, pAKT, and pERK indicate the phosphorylated form of EGFR, AKT, and ERK, respectively. Actin was used as a loading control.

Furthermore, to confirm that the sensitivity/resistance profile data from Ba/F3 stable cells are also observed in *EGFR* mutated human lung cancer derived cell lines, we performed MTS assay with or without EGFR-TKIs using human lung cancer derived cell lines (Figure 4A). The lung cancer cell lines used included PC-9 (*EGFR* exon 19del), H3255 (*EGFR* L858R), PC-9ER (*EGFR* exon 19 del+T790M), and H1975 (*EGFR* L858R+T790M).

The IC50 values of lung cancer cell lines are summarized in Table 1. For cell lines harboring classic *EGFR* mutations, exon 19 deletions (PC-9) and L858R (H3255), afatinib showed the most dramatic inhibitory effect. The calculated IC50 values of afatinib for PC-9 and H3255 were 0.8 nM and 0.3 nM, respectively. Although less potent than afatinib, erlotinib effectively inhibited the proliferation of lung cancer cells as previously described [20]. The calculated IC50 values of erlotinib for PC-9 and H3255 were 7 nM and 12 nM, respectively. The potency of osimertinib was comparable to that of erlotinib. However, rociletinib was less potent than erlotinib and osimertinib. The calculated IC50 values of osimertinib for PC-9 and H3255 were 17 nM and 4 nM, respectively. The calculated IC50 values of rociletinib for PC-9 and H3255 were 84 nM and 35 nM, respectively.

**Table 1:** IC50 values (nM) of lung cancer cell lines

	erlotinib	afatinib	rociletinib	osimertinib
PC-9 (exon 19del)	7	0.8	84	17
H3255 (L858R)	12	0.3	35	4
PC-9ER (exon 19del+T790M)	>10000	165	37	13
H1975 (L858R+T790M)	1185	57	23	5
BID007 (A763_Y764insFQEA)	45	8	1278	40

Erlotinib did not inhibit the proliferation of cell lines harboring *EGFR* T790M, PC-9ER and H1975, at low concentrations. However, afatinib and 3^rd^ generation EGFR-TKIs, osimertinib and rociletinib, effectively inhibited the proliferation of lung cancer cells. The calculated IC50 values of afatinib, osimertinib, and rociletinib for PC-9ER were 165 nM, 13 nM, and 37 nM, respectively. The calculated IC50 values of afatinib, osimertinib, and rociletinib for H1975 were 57 nM, 5 nM, and 23 nM, respectively. These data indicate that the sensitivity/resistance profile data observed in Ba/F3 stable cells are similar to those observed in human lung cancer cell lines.

Furthermore, immunoblotting was performed to determine whether the aforementioned sensitivity of lung cancer cells to EGFR-TKIs was mediated through inhibition of the EGFR signaling pathway (Figure 4B). Consistent with the results of the MTS assay, afatinib most potently inhibited the phosphorylation of EGFR and downstream proteins, AKT and ERK, in PC-9 and H3255 cells. Although less potent than afatinib, erlotinib and osimertinib effectively inhibited the phosphorylation of EGFR, AKT, and ERK in PC-9 and H3255. For the lung cancer cells harboring *EGFR* T790M, all EGFR-TKIs, but erlotinib, effectively inhibited the phosphorylation of EGFR, AKT, and ERK.

In summary, these data indicate that the sensitivity/resistance profile observed in Ba/F3 cells was confirmed in human lung cancer cells.

### Application of the *in vitro* model to *EGFR* exon 20 insertion mutations

Except for *EGFR* A763_Y764insFQEA, most of *EGFR* exon 20 insertion mutations are reported to be resistant to 1^st^ generation EGFR-TKIs. Recently, the results of a clinical trial assessing afatinib treatment of patients with advanced NSCLC harboring uncommon EGFR mutations, including exon 20 insertion mutations have been reported [44]. Of the 23 NSCLC patients harboring *EGFR* exon 20 insertion mutations, only two (8.7%) patients presented an objective response, indicating the limited efficacy of afatinib for exon 20 insertion mutations.

**Figure 5:**
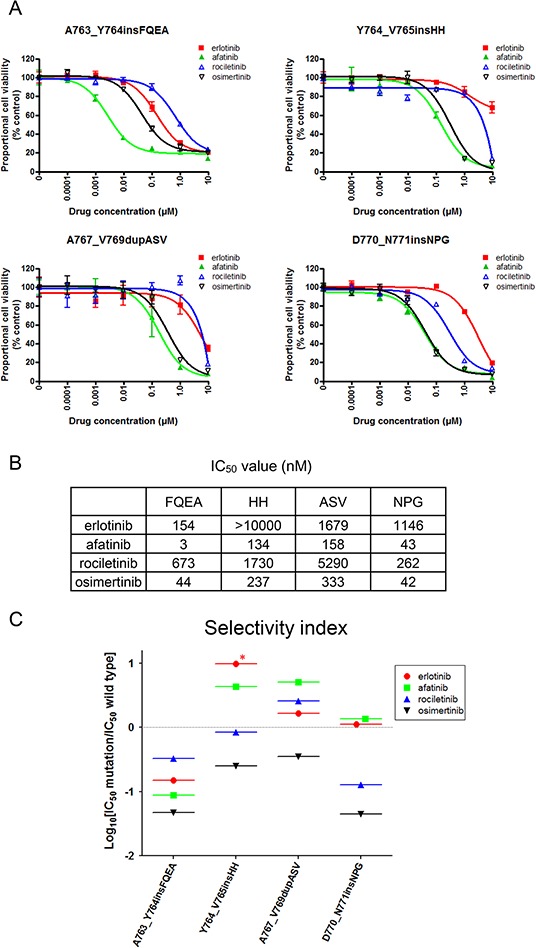
Sensitivity of Ba/F3 cells harboring *EGFR* exon 20 insertion mutations to EGFR-TKIs **A.** MTS assay for Ba/F3 cells harboring *EGFR* exon 20 insertion mutations. The mutations studied include A763_Y764insFQEA, Y764_V765insHH, A767_V769dupASV, and D770_N771insNPG. The proportional cell viability is shown. Erlotinib, afatinib, osimertinib, and rociletinib were used as EGFR-TKIs. Error bars indicate standard deviation. **B.** IC50 values (nM) of EGFR-TKIs for *EGFR* exon 20 insertion mutations. **C.** The calculated values of the selectivity index (SI) for *EGFR* exon 20 insertion mutations are shown. *; SI index > 1.

Until now, there is no EGFR-TKI reported to be effective against exon 20 insertion mutations. To examine the sensitivity/resistance profile of *EGFR* exon 20 insertion mutations to EGFR-TKIs, we performed MTS assays with or without EGFR-TKIs using cells harboring four representative *EGFR* exon 20 insertion mutations, namely, A763_Y764insFQEA, Y764_V765insHH, A767_V769dupASV, and D770_N771insNPG (Figure 5A). Afatinib potently inhibited the growth of cells harboring *EGFR* A763_Y764insFQEA. Of the other three EGFR-TKIs, osimertinib most effectively inhibited Ba/F3 cell growth.

Interestingly, for other 1^st^ generation EGFR-TKIs resistant *EGFR* exon 20 insertion mutations, osimertinib and afatinib presented similar efficacy. The IC50 values of afatinib for Y764_V765insHH, A767_V769dupASV, and D770_N771insNPG were 134, 158, and 43 nM, respectively. The IC50 values of osimertinib for Y764_V765insHH, A767_V769dupASV, and D770_N771insNPG were 237, 333, and 42 nM, respectively (Figure 5B).

To estimate the therapeutic window of EGFR-TKIs for *EGFR* exon 20 insertion mutations, we applied the aforementioned *in vitro* model to exon 20 insertion mutations (Figure 5C). Surprisingly, we found that osimertinib presented the lowest SI values of around −1, indicating IC50 values for *EGFR* exon 20 insertion mutations about 10 fold lower than those for the wild type EGFR. However, the SI values of other EGFR-TKIs were around or above zero. These data indicate that osimertinib may present a wide therapeutic window and is effective for several exon 20 insertion mutations.

### Biological confirmation of osimertinib efficacy for lung cancer harboring *EGFR* exon 20 insertion mutations

To biologically confirm the aforementioned efficacy of osimertinib for lung cancer harboring *EGFR* exon 20 insertion mutations, further *in vitro* analyses were performed.

**Figure 6:**
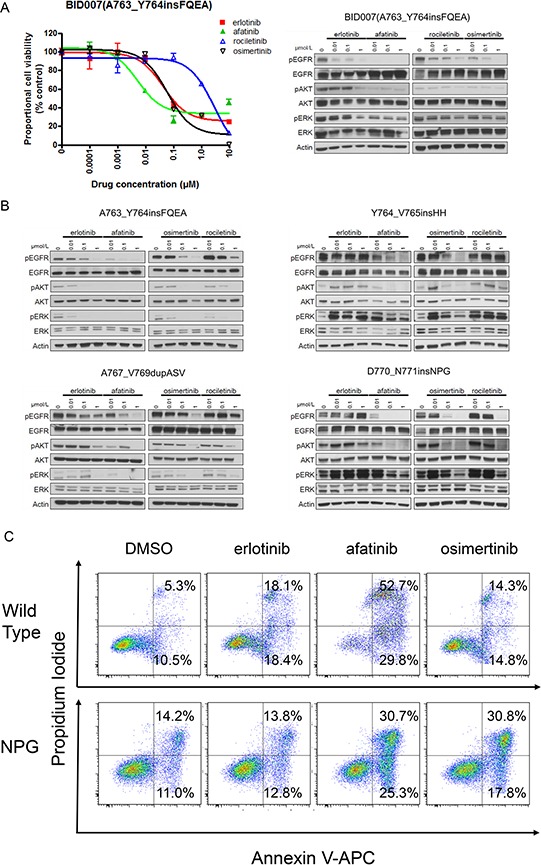
Efficacy of EGFR-TKIs for *EGFR* exon 20 insertion mutations **A.** MTS assay (left) and immunoblotting (right) for BID007 (EGFR A763_Y764insFQEA) cells. Error bars indicate standard deviation. **B.** Results of immunoblotting for Ba/F3 cells with EGFR exon 20 insertion mutations. The cells were treated with the indicated concentrations of EGFR-TKIs for 4 h. Erlotinib, afatinib, osimertinib, and rociletinib were used as EGFR-TKIs. pEGFR, pAKT, and pERK indicate the phosphorylated form of EGFR, AKT, and ERK, respectively. Actin was used as a loading control. **C.** Apoptosis assay using cytometry. Ba/F3 cells harboring wild type *EGFR* and *EGFR* D770_N771insNPG (NPG) were treated with EGFR-TKIs for 48 h, subsequently the cells were stained with propidium iodide and annexin V-APC. The numbers indicate the proportion of annexin V-positive and/or propidium iodide-positive cells.

First, to confirm whether the above sensitivity pattern observed in Ba/F3 cells was also observed in human lung cancer cell lines, we performed immunoblotting and MTS assays using BID007 (*EGFR* A763_Y764insFQEA) cells (Figure 6A). To our knowledge BID007 is the only cell line harboring *EGFR* exon 20 insertion mutations, which we originally established [20]. The sensitivity pattern and inhibition of the phosphorylation of EGFR and downstream proteins were consistent with the results observed in Ba/F3 cells harboring EGFR A763_Y764insFQEA. The most dramatic inhibition of cell growth was observed with afatinib. The calculated IC50 value of afatinib for BID007 was 8 nM. Although less potent than afatinib, erlotinib and osimertinib effectively inhibited the proliferation of BID007 cells. The calculated IC50 value of erlotinib and osimertinib for BID007 was 45 nM and 40 nM, respectively. However, rociletinib was less potent than erlotinib and osimertinib. The calculated IC50 value of rociletinib for BID007 was 1278 nM. These data indicate that the sensitivity pattern observed in Ba/F3 cells was also observed in human lung cancer cell line, BID007.

Next, to confirm that the sensitivity of Ba/F3 cells harboring *EGFR* exon 20 insertion mutations was exerted through inhibition of the EGFR signaling pathway, we performed immunoblotting (Figure 6B). As expected, for *EGFR* A763_Y764insFQEA, afatinib dramatically inhibited the phosphorylation of EGFR, AKT, and ERK. Erlotinib and osimertinib similarly inhibited the phosphorylation of EGFR, AKT, and ERK. For other 1^st^ generation EGFR-TKIs resistant *EGFR* exon 20 insertion mutations, Y764_V765insHH, A767_V769dupASV and D770_N771insNPG, the inhibition of the phosphorylation of EGFR, AKT, and ERK was similar between osimertinib and afatinib.

Finally, to confirm the therapeutic window of osimertinib for 1^st^ generation EGFR-TKIs resistant *EGFR* exon 20 insertion mutations, we performed apoptosis assays using Ba/F3 cells harboring wild type *EGFR* and *EGFR* D770_N771insNPG. We stained the Ba/F3 cells with annexin V-APC and propidium iodide after 48 h of EGFR-TKI treatment (0.1 μM). The proportion of annexin-V positive and/or propidium iodide positive cells was examined by flow cytometry (Figure 6C). As expected, afatinib induced apoptosis in both Ba/F3 cells harboring wild type *EGFR* and *EGFR* D770_N771insNPG, indicating a narrow therapeutic window of afatinib for *EGFR* D770_N771insNPG. The proportion of annexin-V positive cells was 82.5% for the wild type EGFR and 56.0% for *EGFR* D770_N771insNPG. In contrast, the effect of osimertinib on apoptosis in Ba/F3 cells harboring wild type EGFR was low, while it was high in Ba/F3 cells harboring *EGFR* D770_N771insNPG. The proportion of annexin-V positive cells was 29.1% for the wild type EGFR and 48.6% for *EGFR* D770_N771insNPG. These data indicate the wide therapeutic window of osimertinib for *EGFR* D770_N771insNPG.

In summary, we biologically confirmed the efficacy and therapeutic window of osimertinib for *EGFR* exon 20 insertion mutations.

## DISCUSSION

In this study, we compared the potency of erlotinib, afatinib, osimertinib, and rociletinib against multiple types of *EGFR* mutations such as classic *EGFR* mutations, exon 19 deletions and L858R, with or without T790M, or exon 20 insertion mutations. Because EGFR is ubiquitously expressed in epithelial cells, EGFR-TKIs induce toxicity when EGFR-TKIs affect the wild type EGFR in epithelial cells. Hence, the therapeutic window, i.e., the difference in term of the concentrations that affect the wild type and mutated EGFR, is important. In this study, we created an *in vitro* model to determine the therapeutic window of EGFR-TKIs by calculating the ratio of IC50 values of EGFR-TKIs in cells stably expressing the wild type *EGFR* or the mutated *EGFR*.

In general, the data from Ba/F3 stable cell lines and human lung cancer derived cell lines were consistent. For classic *EGFR* mutations, exon 19 deletions and L858R, afatinib was the most potent. Our data also showed a wide therapeutic window of afatinib for these mutations. However, in the clinic, the high frequency of adverse events such as skin rash or diarrhea is repeatedly reported [34, 35], indicating that the *in vivo* concentration of afatinib might exceed the concentration that inhibits the wild type EGFR. It is possible that, by reducing the dose, afatinib retains its ability to inhibit the mutated EGFR, but not the wild type EGFR.

For *EGFR* T790M positive mutations, osimertinib and rociletinib showed a strikingly potent inhibition compared to erlotinib or afatinib. In addition, osimertinib and rociletinib showed a wide therapeutic window for these mutations. These data indicate that osimertinib and rociletinib are effective and safe EGFR-TKIs for *EGFR* T790M positive mutations. In human clinical trials, osimertinib and rociletinib showed promising safety and efficacy, even though some hyperglycemia was observed in patients treated with rociletinib [38, 39].

Interestingly, for 1^st^ generation EGFR-TKIs resistant exon 20 insertion mutations, Y764_V765insHH, A767V769dupASV, and D770_N771insNPG, osimertinib showed a similar potency and higher mutation specificity than afatinib. However, the IC50 values of osimertinib for these exon 20 insertion mutations are 10 to 100 fold higher than those for the classic *EGFR* mutations with or without T790M mutations. Thus, higher concentrations of osimertinib may be necessary to effectively treat the patients with 1^st^ generation EGFR-TKIs resistant exon 20 insertion mutations.

Preliminary pharmacokinetic profile, showing total plasma concentrations of osimertinib, was reported [30]. The mean plasma concentration of 6 patients treated with once-daily 20 mg oral osimertinib dosing in the AURA phase I study (NCT01802632) was around 100 nM, which is around the IC50 values found in this study for 1^st^ generation EGFR-TKIs resistant exon 20 insertion mutations (42–333nM). In addition, the results of this phase I study have been reported [38]. The patients were assigned to once-daily dosing from 20 mg to 240 mg. No dose-limiting toxicity was observed during the 28-day evaluation period at any dose levels. Therefore, a maximum tolerated dose was not determined. The maximum serum concentrations were 106.3, 305.1, 635.4, 1006, and 1510 nM for 20, 40, 80, 160, and 240 mg dose levels, respectively. These data indicate that osimertinib concentration may be adjusted to the level that effectively inhibits the mutant EGFR, but not the wild type EGFR. To examine the efficacy of osimertinib for NSCLC harboring 1^st^ generation EGFR-TKIs resistant exon 20 insertion mutations, dose adjusted clinical trials are anticipated.

From the EGFR-TKIs oriented viewpoint, erlotinib is effective for *EGFR* exon 19 deletions, L858R, and A763_Y764insFQEA. However, erlotinib does not seem to be effective for *EGFR* T790M positive mutations as previously described [20]. Afatinib seems to be effective and has a wide therapeutic window for *EGFR* exon 19 deletion and L858R. However, afatinib was less potent than osimertinib and rociletinib for *EGFR* T790M positive mutations. Osimertinib and rociletinib showed a potent efficacy and a wide therapeutic window for *EGFR* T790M positive mutations.

Even for 3^rd^ generation EGFR-TKIs, the potency spectrum was clearly different between osimertinib and rociletinib. Interestingly, for *EGFR* 19 deletion and L858R, osimertinib efficacy was comparable with that of erlotinib, indicating that osimertinib can be used for *EGFR* 19 deletion and L858R as first line EGFR-TKI. Furthermore, we identified the therapeutic window of osimertinib for 1^st^ generation EGFR-TKIs resistant exon 20 insertion mutations. The reason why osimertinib showed a distinct wide mutation spectrum is unknown. However, it may be partly due to the distinct chemical structure of this compound [30]. To our knowledge, this is the first report, which clearly demonstrates the difference in term of mutation spectrum of EGFR-TKIs against various types of *EGFR* mutations.

However, our study includes only *in vitro* study. The pharmacokinetics and pharmacodynamics may vary among EGFR-TKIs. Hence, EGFR-TKI *in vivo* concentrations may vary among EGFR-TKIs. To further develop a better strategy for using EGFR-TKIs, further *in vivo* and human clinical trials are mandatory.

In summary, we created an *in vitro* model to determine the therapeutic window of EGFR-TKIs, which matched well with the data obtained from human clinical trials. Interestingly, by applying the model, osimertinib showed the widest therapeutic window in relation to wild type EGFR for most mutations including *EGFR* exon 20 insertion mutations. This model will provide a preclinical rationale for proper selection of EGFR-TKIs against clinically-relevant *EGFR* mutations.

## MATERIALS AND METHODS

### Cell lines

Five human NSCLC cell lines were used, namely, PC-9 [*EGFR* exon 19 deletion (delE746-A750)], H3255 [*EGFR* L858R], PC-9ER [*EGFR* exon 19 deletion (delE746-A750)+T790M], BID007 [*EGFR* exon 20 insertion (A763_Y764insFQEA)], and H1975 [*EGFR* L858R+T790M]. PC9 cells were a kind gift from Dr. Pasi Janne (Dana-Farber Cancer Institute, Boston, MA, USA). H3255 and H1975 were purchased from the American Type Culture Collection (Manassas, VA, USA). PC-9ER cells become resistant to erlotinib after chronic exposure to erlotinib through acquisition of *EGFR* T790M second mutation. BID007 was originally established [20]. Cell authentication for H1975 and H3255 was performed in June 2015.

### Reagents

Erlotinib and afatinib were purchased from LC Laboratories (Woburn, MA, USA). Osimertinib and rociletinib were purchased from Selleck Chemicals (Houston, TX, USA). Total EGFR antibody (#2232), total AKT antibody (#9272), phospho-AKT (S473; D9E) antibody (#4060), total p44/42 MAPK antibody (#9102S), and phospho-p44/42 MAPK (T202/204) antibody (#9101S) were purchased from Cell Signaling Technology (Beverly, MA, USA). Phospho-EGFR (Y1068) antibody (44788G) was purchased from Invitrogen/Life Technologies (Carlsbad, CA, USA). Actin antibody was purchased from Sigma-Aldrich (St. Louis, MO, USA).

### Ba/F3 stable cell lines

Ba/F3 cells stably expressing the wild type and mutated *EGFR* were created as previously described [20]. Ba/F3 cells harboring *EGFR* mutations were cultured in RPMI-1640 growth medium, supplemented with 10% fetal bovine serum at 37°C in a humidified 5% CO_2_ incubator. Ba/F3 cells expressing *EGFR* wild type were cultured in RPMI-1640 growth medium, supplemented with 10% fetal bovine serum at 37°C in a humidified 5% CO_2_ incubator with EGF (10 ng/mL). The *EGFR* mutations examined in this study include delL747_P753insS (exon 19del), L858R, delL747_P753insS+T790M (exon 19del+T790M), L858R+T790M, A763_Y764insFQEA, Y764_V765insHH, A767_V769dupASV, and D770_N771insNPG.

### Cell proliferation assay

The MTS assay was performed as previously described [20]. PC-9, H3255, PC-9ER, H1975, and BID007 were seeded in 96-well plates. Twenty-four hours after seeding, the appropriate medium with or without EGFR-TKI was added to each well. Control cells were treated with the same concentration of the vehicle, dimethyl sulfoxide (DMSO). Seventy-two hours after treatment, absorbance was measured.

For Ba/F3 cells, the cells were seeded with or without EGFR-TKI. Seventy-two hours after seeding, absorbance was measured. All experiments were performed at least three times.

### Immunoblotting analysis

Cells were treated with EGFR-TKI at concentrations of 0.01–1 μmol/L for 4 h. Cells were lysed in Cell Lysis Buffer (Cell Signaling Technology). Equal amounts of protein were loaded per lane on sodium dodecyl sulfate-polyacrylamide gels. Separated proteins were transferred to polyvinylidene fluoride membranes. The membranes were incubated overnight with primary antibodies at 4°C and then incubated with secondary antibodies for 1 h. For the detection of proteins, the membranes were incubated with agitation in LumiGLO reagent and peroxide (Cell Signaling Technology) and then exposed to X-ray film.

### Apoptosis assay

Ba/F3 cells harboring wild type *EGFR* and *EGFR* D770_N771insNPG were seeded in 6-well plates. The cells were treated with EGFR-TKIs (0.1 μM) for 48 h. Control cells were treated with the same concentration of the vehicle, DMSO. We analyzed the apoptotic status of cells using the Annexin V Apoptosis Detection Kit APC (eBioscience, San Diego, CA, USA) according to the manufacturer's protocol. The proportion of apoptotic cells was evaluated by flow cytometric analysis, using the Gallios flow cytometer system (Beckman Coulter, Brea, CA, USA).

### Statistical analysis

Statistical analysis was performed using the GraphPad Prism software, version 4.0 (GraphPad Software, La Jolla, CA, USA). IC_50_ was calculated by using the GraphPad Prism software.
